# Emergency Care Needs in an Asymmetric Conflict: A Facility‐Based Cohort Study From Israel on October 7, 2023

**DOI:** 10.1002/wjs.70516

**Published:** 2026-08-01

**Authors:** Maximilian P. Nerlander, Mohammad Abu Issa, Mor Saban, Gili Givaty, Yaniv Ovadia

**Affiliations:** ^1^ Center for Disaster Medicine and Traumatology Linköping University Linköping Sweden; ^2^ Hebrew University of Jerusalem Braun School of Public Health and Community Medicine Jerusalem Israel; ^3^ Emergency Department Barzilai University Medical Center Ashkelon Israel; ^4^ Department of Nursing Sciences Gray Faculty of Medical and Health Sciences Tel Aviv University Tel Aviv Israel; ^5^ Rehabilitation Wing Ministry of Defense Petach Tikva Israel; ^6^ Big Data Unit Barzilai University Medical Center Ashkelon Israel

**Keywords:** armed conflict, disaster medicine, emergency department, injury epidemiology, prehospital death

## Abstract

**Background:**

Due to the deteriorating global security situation, many countries are preparing their health systems for armed conflict. Wartime health care delivery is challenging due to the loss of territorial control and attacks on Emergency Medical Services (EMS). The objectives of this study were to use Emergency Department (ED) data from the hospital closest to the areas of fighting during the October 7, 2023, attacks in Israel, to determine what patient factors relate to status as dead on arrival (DoA), high acuity, and to explore the epidemiology of injury patterns.

**Methods:**

This is a retrospective cross‐sectional cohort study. All patients presenting with injuries from the October 7, 2023, attacks, from 06h30 until 24h00, were included. Variables included demographics, patient classification (civilian or combatant), mode of arrival, condition, injury mechanism and injury location. Two logistic regression models with backward elimination were used to determine the patient factors associated with DoA and high acuity.

**Results:**

A total of 301 patients were included. A majority (67.8%) arrived at the ED informally instead of by ambulance (23.9%). A total of 26.6% were DoA. Informal arrival was associated with DoA (OR 10.73, CI [4.18–36.55]). Injuries by firearms and thoracic injuries had higher odds of high acuity (OR 4.15 95% CI [1.67–11.50] and OR 4.35 95% CI [1.49–12.95]), while extremity injuries had lower odds (OR 0.41 95% CI [0.17–0.99]). Firearm‐related injuries were more common among combatants compared to civilians (55.3% vs. 35.1%, *p* *≤* 0.05). Extremity injuries accounted for more than half of injuries in both civilians and combatants (52.3% vs. 59.5%, *p* = 0.31).

**Conclusion:**

In an armed conflict with loss of territorial control and reduced EMS access, healthcare planners may need to anticipate significant prehospital death and that many patients arrive at hospital informally. Prehospital death may be reduced by expanding lay bystander skills in trauma care as these may access patients before ambulances do. Due to survival bias, patients with firearm‐related injuries may have more urgent care needs upon reaching hospital than patients with explosion‐related injuries. Given the higher survivability of extremity injuries, these may be overrepresented among patients who reach hospital.

## Graphical abstract

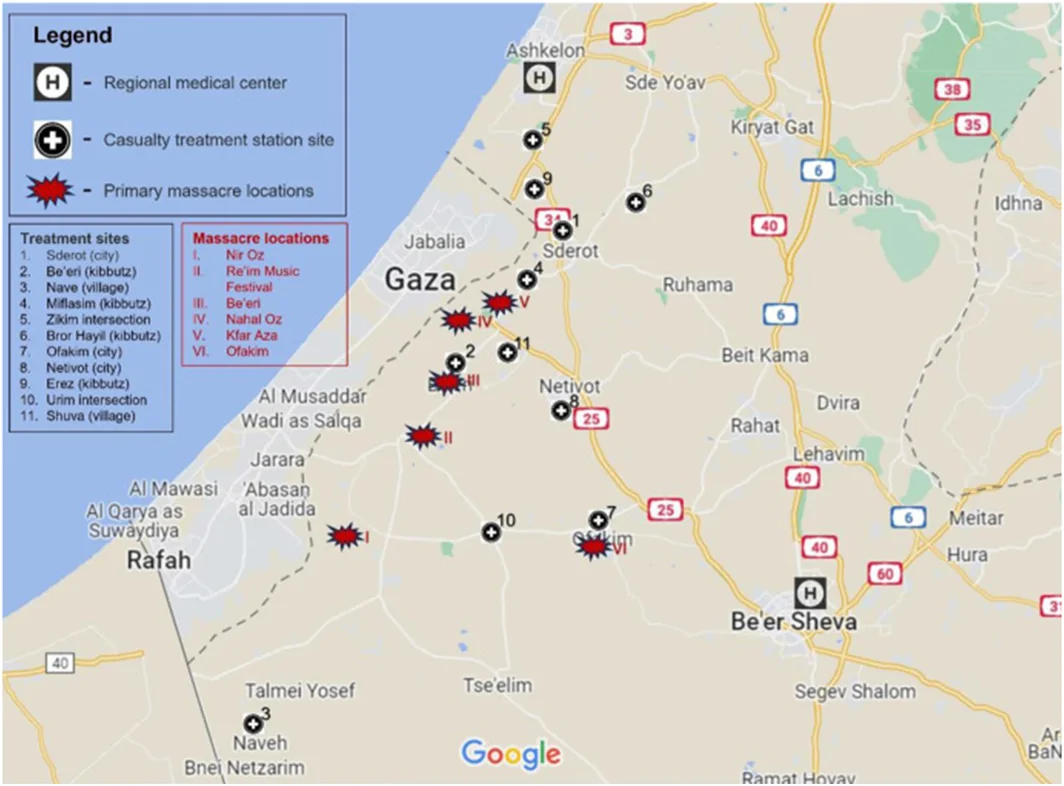

## 1 Introduction

Armed conflict is a pervasive threat to health worldwide. The Norway‐based Peace Research Institute Oslo estimates that in 2025, there were approximately 255,000 deaths from armed conflict, driven mainly by the wars in Ukraine, Sudan and Gaza [1]. Throughout the Cold War, western countries had well‐developed preparedness measures to maintain health system function in case of war, which were subsequently deconstructed following the dissolution of the Soviet Union. The 2022 Russian full‐scale invasion of Ukraine has created an urgent need to reintroduce and update these measures, especially in Europe and North America [2, 3, 4]. However, cold‐war era preparedness measures may be obsolete in preparing trauma systems for the challenges of modern armed conflict. The asymmetric tactics seen in many modern theaters, including attacks on health care assets and the targeting of civilians, pose unique challenges to healthcare delivery. Since most armed conflicts occur in low‐ or middle‐income countries with weak prewar health care systems, the conclusions drawn from existing studies from these contexts may not be generalizable to health care systems in high‐income countries [5, 6, 7].

Israel, while geographically a small country, has a health care system with similar resources and quality to other developed nations [8]. Therefore, studies from the Israeli health care system during the October 7, 2023, attacks are likely to generate evidence which is generalizable to preparedness efforts in other high‐income settings. In Israel, Magen David Adom (MDA) is the nation's Emergency Medical Service (EMS) and a member of the International Red Cross and Red Crescent Movement. It operates through a nationwide network of community‐based volunteer first responders, Emergency Medical Technicians (EMTs) and salaried paramedics. In case of a dispatch call, nearby volunteers are alerted and travel rapidly to the patient, usually by motorbikes, before other assets arrive. These first responders carry a compact trauma kit which contains tourniquets and hemostatic dressings which enable them to stabilize the patient at point of injury. In parallel, higher tiers of prehospital resources are activated including Basic Life Support (BLS) and Advanced Life Support (ALS) ambulances. BLS ambulances are staffed by an EMT and volunteer first responders while ALS ambulances are staffed by paramedics. In total, MDA has approximately 500 medical teams operating out of 200 ambulance stations in Israel [9]. Nationwide, Israel has nine local trauma centers, 13 regional trauma centers and seven supra‐regional trauma centers. These designations are issued by the Israeli Ministry of Health (MoH) and approximate to levels III, II and I trauma centers in the United States [10].

Due to their multimodal nature and widespread scale, the attacks on October 7, 2023, in Israel provide an example of the asymmetric warfare tactics that characterize modern conflicts and for which many countries now prepare [11]. The attacks commenced with a rocket barrage which triggered air raid sirens and the Iron Dome air defense system, which detects and shoots down incoming rockets [12]. This was followed by a ground invasion by approximately 3000 militants. Throughout the day, an increasing area of southern Israel came under the control of the aggressors. Over 800 square kilometers were affected, including 55 civilian communities and the larger towns of Ofakim and Sderot. The provision of health care on October 7 saw many of the challenges seen in the war in Ukraine [13, 14, 15]. The ongoing rocket barrage created hazardous conditions for staff to travel to hospital, hospitals and EMS came under attack, and the resulting prehospital access constraints resulted in many patients arriving to hospital by informal means rather than by ambulance. Israel, similar to many European countries, does not have dedicated military hospitals, and large numbers of civilians and combatants were therefore treated in the same emergency care system. October 7 represents the first historical instance where this type of large‐scale asymmetric attack takes place in a high‐income country. There are several evidence gaps from this setting. These include how prehospital access constraints influence prehospital mortality, what factors are associated with high acuity in the Emergency Department (ED), and what injury patters are seen in patients who reach the ED alive. An expanded understanding of these aspects will add to the knowledge base needed to meet the unique healthcare needs arising from asymmetric warfare tactics.

This study utilized data from the hospital closest to the frontline on October 7 and had two specific objectives. First, to determine which factors were associated with status as dead on arrival (DoA) and high acuity. Second, among patients who arrived at hospital alive, to describe the epidemiology of injury patterns and to investigate if these differed according to patient status as combatant or civilian and by mode of arrival.

## 2 Methods

### 2.1 Study Setting

**FIGURE 1:**
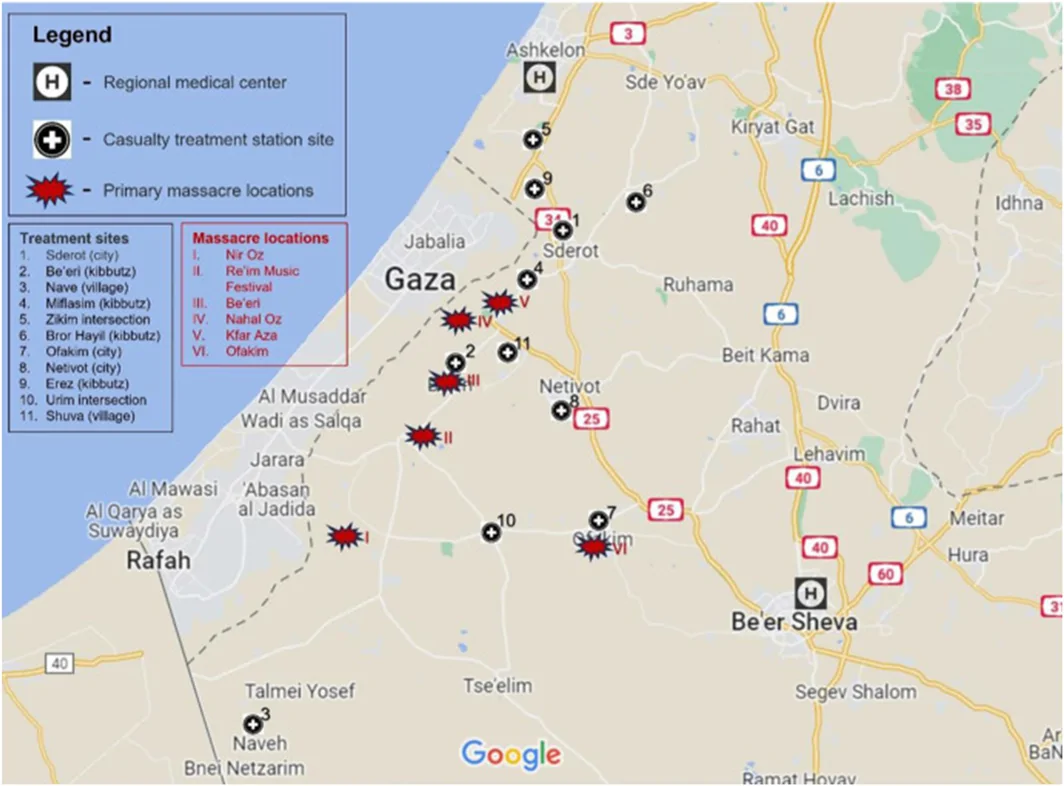
Areas of fighting, trauma stabilization points (casualty treatment stations), and hospitals (regional medical centers) on October 7, 2023. Copyright Eli Jaffe. Reproduced with permission [9].

On October 7, MDA had not anticipated a scenario where they would be denied access to patients on Israel's territory due to their personnel and assets coming under attack. Therefore, MDA mobilized up to 10 armored ambulances to enter the hostile areas and pick up patients who were then transported to a network of Trauma Stabilization Points (TSPs) that were set up adjacent to the areas of fighting. Approximately 100 rounds were made by these ambulances, and 306 patients were treated at the TSPs. From there, patients would be taken to hospital by regular ambulances after stabilization. In a minority of cases, patients would be transported straight to hospital from the scene of injury by the armored ambulances [9]. (Figure 1)

The majority of injured patients presented to the two hospitals closest to the fighting: Barzilai University Medical Center (BMC), Ashkelon, 10 km from the northern border of Gaza, and Soroka Medical Center (SMC), Beer‐Sheba, 40 km from the eastern border of Gaza. These received a total of 323 and 673 patients respectively during the 24h‐period following the start of the attacks [16]. Smaller numbers of patients were seen in more distant hospitals north of Ashkelon, including Rehovot (98), Beer Yakov (87) and Ashdod (87) [17].

### 2.2 Study Design and Data Sources

This is a cross‐sectional cohort study utilizing routinely collected registry data from patients treated at the ED at BMC, which is a 660‐bed regional trauma center [18, 19]. The ED collects patient data according to a standardized data collection tool. Among patients admitted to the wards, there was no mechanism to retroactively populate the ED registry with data that is generated at later stages during the hospital stay. Therefore, only registry data collected at ED level were included in the study. Data were extracted from 06h30 until 24h00 on October 7, 2023, as this time frame represented the peak loss of territorial control during the attacks [20]. All patients presenting to the ED with conditions sustained during the October 7 attacks were included. Patients with conditions unrelated to the attacks were excluded (*n* = 4).

The following variables were used for the study.

1. Demographics: Age and Sex
2. Arrival time: hh:mm:ss.
3. Patient classification: Civilian or Combatant. Combatants were defined as members of the Israel Defense Forces (IDF) or police based on identity documents. Police are generally not considered combatants under international humanitarian law. However, the conditions of October 7 resulted in police being injured under combat‐like circumstances and were subsequently eligible for official recognitions, including medals that are normally reserved for soldiers. Further, police have access to weapons, body armor and vehicles not generally available to civilians. Thus, for the purpose of the analysis, injured police officers were grouped together with members of the IDF.
4. Mode of arrival: Ambulance or Informal arrival. The latter included private vehicle, public transport, or by foot.
5. Condition: Mild (ISS 1–9), Moderate (ISS 9–15), Severe (ISS 16–24), Critical (25+), Anxiety (patients with no evidence of physical injuries), or DoA (patients arriving with no signs of life). Following a mass casualty incident, this variable is retroactively assessed for MoH reporting purposes and describes the patient's condition in the ED. It is based on a combination of the patient's ISS together with a physician‐led chart review of relevant clinical findings and vital signs. ISS score correlation is not absolute, and final level is assigned by the reviewing physician based on clinical judgment.
6. Injury mechanism: Firearm, Explosion, Siren, Iron Dome, Other, Combined. Siren is defined as fall injury from running to shelter during air raid siren activation. Iron Dome is defined as injuries sustained from Iron Dome shrapnel. The Other category include infrequent and miscellaneous injuries including burns, stabs, and blunt injuries. Combined mechanism is defined as two or more of the aforementioned mechanisms.
7. Injury location: Head, Face/neck, Thorax, Back, Abdomen, Pelvis, Extremities, > 1 injury location, Other.

### 2.3 Statistical Analyses

The primary outcomes were DoA and high acuity. DoA was defined as patients presenting without signs of life and declared deceased upon initial assessment (DoA = 1 or 0). High acuity was defined as patients determined to be severe or critical (severe or critical = 1; all other categories = 0). Two logistic regression models with backward elimination were used: In Model 1, DoA was the dependent variable. Explanatory variables included sex, classification, and mode of arrival. Due to the operational constraints posed by the attacks, there were no provisions to collect additional variables in this patient group. In Model 2, high acuity was the dependent variable. Explanatory variables included sex, patient classification, mode of arrival, injury location and injury mechanism. For injury mechanism, dummy variables were created for each separate mechanism. Another separate variable was created to directly compare firearms and explosions as these were the two predominant mechanisms. In both models, patients presenting with only anxiety were excluded from the analysis as this subset of patients were not physically injured.

The secondary outcomes were the proportions of injury mechanisms and locations of injury stratified according to patient classification and mode of arrival. Continuous variables were described using median and interquartile ranges. Categorical variables were summarized using proportions. Fisher's exact test was used to compare strata. A *p*‐value of < 0.05 was considered significant throughout. Missing data were removed from the denominator. Data were collected in Microsoft Excel and analyzed using JMP 13 (SAS Institute Inc., SAS Campus Drive, Cary, North Carolina 27,513). This study adhered to the Strengthening the Reporting on Observational Studies in Epidemiology (STROBE) guidelines [21]. Ethical approval was granted by the BMC Helsinki Committee (BRZ‐0083‐24) with a waiver of informed consent. Ethical approval was also granted by the Swedish National Ethical Review Board (2026‐00449‐02).

## 3 Results

A total of 301 patients were included (Table 1). Most were male (78.1%), and the median age was 30 years. Almost two thirds of patients were civilian (61.1%). A majority (67.8%) arrived at the ED informally rather than by ambulance (23.9%). A total of 80 patients (26.6%) were DoA. Moderate condition accounted for the highest proportion (36.5%), while smaller proportions of patients were severe (11.0%) or critical (1.3%). Firearms and explosions accounted for the majority of injuries (42.3% and 38.0% respectively), while one‐tenth of injuries were siren‐ or Iron Dome‐related (7.7% and 2.4% respectively).

**TABLE 1:** Characteristics of the study population and patient condition in the emergency department, *n* = 301.

Total (*n*, %)	301 (100)
Sex (*n*, %)	
Male	235 (78.1)
Female	66 (21.9)
Missing values (*n*)	0
Age (median, [IQR])^a^	30 [21–45]
Missing values (*n*)	80
Classification (*n*, %)	
Civilian	184 (61.1)
Combatant	117 (38.9)
Missing values (*n*)	0
Mode of arrival (*n*, %)	
Ambulance	72 (23.9)
Informal	204 (67.8)
Other/unknown	25 (8.3)
Missing values (*n*)	25
Condition in ED (*n*, %)	
Dead	80 (26.6)
Critical	4 (1.3)
Severe	33 (11.0)
Moderate	110 (36.5)
Mild	61 (20.3)
Anxiety	8 (2.7)
Unknown/other	5 (1.7)
Missing values (*n*)	6
Injury mechanism^a^ ^,^ ^b^	
Explosion	79 (38.0)
Firearm	88 (42.3)
Siren	16 (7.7)
Iron dome	5 (2.4)
Other	13 (6.3)
Combined	7 (3.4)
Missing values (*n*)	12

^a^ Excludes patients who were dead on arrival for whom age and injury mechanism were not recorded (*n* = 80).

^b^ Excludes Anxiety (*n* = 8).

Tables 2 and 3 depict the results of the two regression models. In Model 1, only mode of arrival was associated with status as DoA (OR 10.73, CI [4.18–36.55]). No statistically significant relationship was found between DoA and sex and classification (Table 2). In Model 2, firearm‐related injuries and thoracic injuries were independently associated with an increased risk of high acuity (OR 4.15 95% CI [1.67–11.50] and OR 4.35 95% CI [1.49–12.95] respectively). Firearm‐related injuries were only significantly associated with high acuity when explosion‐related injuries were used as reference and less common injuries were excluded. Injuries to the extremities had a decreased risk (OR 0.41 95% CI [0.17–0.99]). There was no statistically significant relationship between high acuity and sex, mode of arrival, classification, and remaining injury mechanisms and injury locations (Table 3).

**TABLE 2:** Patient factors associated with dead on arrival status.

Factor	Reference	Odds ratio	*p*‐value	95% confidence intervals	
Informal	Ambulance	10.73	< 0.05	4.18	36.55
Combatant	Civilian	1.53	0.16	0.85	2.73
Male	Female	1.77	0.11	0.88	3.72

**TABLE 3:** Patient factors associated with high acuity.

Factor	Reference	Odds ratio	*p*‐value	95% confidence intervals	
Firearm	Explosion	4.15	< 0.05	1.67	11.50
Thorax	No thoracic injury	4.35	< 0.05	1.49	12.95
Extremity	No extremity injury	0.41	< 0.05	0.17	0.99

Table 4 depicts the injury characteristics of the study population. A smaller proportion of civilians than combatants were injured by firearms (35.1% vs. 55.3%, *p* ≤ 0.05) while proportions of injuries from explosions were similar (37.8% vs. 36.4%, *p* = 0.77). Siren‐ or Iron Dome‐related injuries almost exclusively occurred among civilians (11.5% vs. 1.3%, *p* ≤ 0.05% and 3.8% vs. 0.05, *p* ≤ 0.05 respectively). Injuries from explosions were less common among ambulance arrivals compared to informal arrivals. However, this relationship was not statistically significant (30.8% vs. 42.9%, *p* = 0.11). The proportions of firearm‐related injuries were similar across informal and ambulance arrivals (44.6% vs. 40.3%, *p* = 0.57).

**TABLE 4:** Injury mechanism and anatomical location of injury, *n* = 213.

	By classification			By mode of arrival		
	Civilian	Combatant	*p*‐value	Ambulance	Informal	*p*‐value
Injury mechanism (*n*, %)^a^						
Firearm	46 (35.1)	42 (55.3)	< 0.05	29 (44.6)	48 (40.3)	0.57
Explosion	51 (38.9)	28 (36.8)	0.77	20 (30.8)	51 (42.9)	0.11
Siren	15 (11.5)	1 (1.3)	< 0.05	8 (12.3)	8 (6.7)	0.20
Combined	4 (3.1)	3 (4.0)	0.073	3 (4.6)	3 (2.5)	0.45
Other	10 (7.6)	2 (2.6)	0.14	2 (3.1)	8 (6.7)	0.30
Iron dome	5 (3.8)	0 (0.0)	< 0.05	3 (4.6)	1 (0.8)	0.09
Missing (*n*)	4	5	—	2	6	—
Injury location (*n*, %)^a^						
Head	23 (17.4)	8 (10.0)	0.14	11 (16.4)	18 (14.9)	0.78
Face/Neck	13 (9.9)	7 (8.8)	0.79	7 (10.5)	11 (9.1)	0.09
Thorax	23 (17.3)	6 (7.6)	0.05	8 (11.9)	19 (15.7)	0.48
Abdomen	18 (13.5)	8 (10.1)	0.47	10 (14.9)	14 (11.6)	0.51
Back	17 (12.9)	3 (3.8)	< 0.05	7 (10.5)	12 (10.0)	0.92
Pelvic	8 (6.1)	8 (10.1)	0.30	4 (6.0)	9 (7.5)	0.69
Extremity	69 (52.3)	47 (59.5)	0.31	35 (52.2)	69 (57.5)	0.49
> 1 region	19 (14.1)	8 (9.9)	0.37	10 (14.9)	14 (11.2)	0.46
Other	5 (3.8)	7 (8.9)	0.12	2 (3.0)	7 (5.8)	0.39
Missing (*n*)	21	14	—	0	35	—

^a^ Excludes patients who presented with anxiety (*n* = 8), and those who were dead on arrival (*n* = 80). Injury mechanism and location of injury were not recorded in the latter group.

Extremity injuries accounted for more than half of injuries among both civilians and combatants (52.3% vs. 59.5%, *p* = 0.31). Injuries to the back were significantly more common among civilians (12.9% vs. 3.8%, *p* ≤ 0.05%). Similarly, thoracic injuries were more common among civilians, with a borderline statistical significance (17.3% vs. 7.6%, *p* = 0.05). There were no statistically significant differences in other injury locations between the two groups. No significant differences in injury location were found between informal and ambulance arrivals.

## 4 Discussion

This study aimed to investigate the factors associated with DoA, high acuity, and to describe how the epidemiology of injury patterns differed according to patient classification and mode of arrival. A quarter of patients were DoA, with a strong association with informal arrival. Firearm‐related injuries and thoracic injuries were associated with high acuity. Explosion‐related injuries and injuries to the extremities predominated, with firearm‐related injuries being more common among combatants.

The high proportion of DoA may reflect that unstable but potentially salvageable patients did not reach care in time. This is supported by only a small minority of patients being critical or severe, indicating that a minority of unstable patients reached hospital before deteriorating. In contrast to BMC, out of the 673 patients seen at SMC in Beer‐Sheba, only 1.6% were DoA [16]. Given the greater distance to SMC from the areas of fighting, those who transported critically wounded patients may have prioritized driving to BMC as this was geographically closer. Comparable observations were made in studies of two hospitals that received trauma patients from the 2016‐17 battle of Mosul; these were located at 30 and 90 km from the frontline and saw in‐hospital mortalities of only 0.5%, indicating that loss of EMS access and distance caused unstable but potentially salvageable patients to not reach care in time [5, 22].

There are two potential explanations for the higher odds of DoA status among informal arrivals. First, lack of knowledge or equipment for bystanders to stabilize patients at the scene of injury or en route to hospital. Second, patients who were dead or non‐salvageable may have been inappropriately transported to hospital by bystanders. Given the limited variables collected in patients who were DoA, along with a lack of prehospital information on whether or not the patients had signs of life prior to transport, the data in this study cannot demonstrate whether or not a patient who was DoA was potentially salvageable. While a subset of this group may have been preventable deaths, this remains speculative and unverifiable. The much lower odds of DoA among ambulance arrivals is likely a consequence of the MDA strategy of setting up TSPs. According to the organization, 98 patients were treated in the five TSPs located in the proximity of BMC, allowing for unstable patients to be sufficiently stabilized to survive transport to hospital [9]. Further, given that the MDA chain of care was conducted by trained paramedics, this would have prevented inappropriate transport of dead or non‐salvageable patients. At BMC, patients who were DoA represents a group that largely arrived at hospital outside this chain of care.

Prehospital trauma survival depends on immediate bystander response when EMS is unavailable. Strengthening lay person trauma stabilization skills may therefore be an important health system preparedness measure in asymmetric warfare scenarios where EMS access is negated. Training concepts such as *Stop the Bleed*, which teaches hemostasis at the point of injury, including the use of tourniquets, and *The First 7 Minutes*, which is a course developed by MDA to teach bystanders to administer a mass casualty scene, may be valuable to build this type of capacity [23, 24]. Tourniquets have been demonstrated as safe and effective in western military and civilian systems where patients are rapidly evacuated to definitive care [25, 26, 27, 28, 29, 30]. However, as described in Ukraine, tourniquets carry their own inherent risks in case of prolonged evacuation times, including ischemic damage and the development of Prolonged Tourniquet Application Syndrome [31, 32, 33, 34]. Thus, measures to expand tourniquet use in a conflict‐prone civilian population must be weighed against these risks. It is essential that implementation measures are combined with sufficient training for bystanders to be skilled at identifying correct indications for tourniquet placement, and at reassessing the tourniquet in case of prolonged evacuation times.

The analysis revealed an increased risk of high acuity in thoracic and firearm‐related injuries. In thoracic injuries, the observation is biologically plausible as these injuries endanger the heart, lungs, and great vessels. The lower odds of high acuity seen in extremity injuries may similarly reflect that injuries to these structures are less likely to cause systematic decompensation. By contrast, the observation that firearm‐related injuries had a greater risk of high acuity is contradictory to existing literature on firearm‐ and explosion‐related injuries, where the latter's likelihood of causing severe and complex injuries is well documented [35, 36, 37, 38, 39]. The high proportion of patients who were dead on arrival indicate extensive survival bias in this study. Critical patients with explosion‐related injuries may not have reached care in time or been left in the field as expectant. Thus, a paradoxical pattern is seen in this study where patients with firearm‐related injuries reach care in time and account for comparatively more severe injuries at ED level. From a preparedness perspective, medical facilities may consider this phenomenon in preparing to meet health care needs in armed conflicts with disrupted EMS access and resultant extensive prehospital mortality.

Among patients who reached hospital alive, explosions accounted for less than half of injuries, with similar proportions in both civilians and combatants. Firearm‐related injuries were more common among combatants than civilians. Similarly, among the 673 patients treated at SMC, less than half of injuries were due to explosions [16]. The predominance of firearm‐related injuries among combatants may be due to these being armed themselves, which would have negated the ability of the aggressors to come within reach for some hand‐held explosives to be an effective weapon. Further, a documented tactic on October 7 included throwing explosives into shelters where many civilians had taken cover against the rockets [40]. Since confined spaces potentiate the effects of explosives, this tactic may therefore have increased the proportion of explosion‐related injuries among civilians. By contrast, a 2020 review by Wilde et al. of 49 reports from armed conflicts in 18 countries, which included 58,578 patients, revealed that among civilians and local combatants (defined as non‐Western military in the study), explosions accounted for 50.2% of injuries, which is a higher proportion than demonstrated in this study. Similar to the paradoxical observation of higher acuity in firearm‐related injuries, these may be artifacts of extensive survival bias in this study which distorts the relative proportions of firearm‐ and explosion‐related injuries.

One tenth of patients presented with injuries from running to shelter during air raid sirens, or from Iron Dome shrapnel. Similarly, a study using nationwide Israeli EMS data demonstrated that during the 2023 Israel‐Hamas war which followed October 7, there were 636 dispatches related to seeking shelter, with a predominance of orthopedic injuries, head injuries and anxiety attacks [41]. These may be significant observations to inform wartime preparedness; many countries, especially in Europe, are scaling up their air raid shelter infrastructure and air defenses. However, the results in this study demonstrate that these carry their own risks [42].

Most injuries occurred in the extremities regardless of patient classification, and this relationship was also seen at SMC, where the extremities accounted for 70% of injuries [16]. A study from the Israeli National Trauma Registry demonstrated a similar predominance in the October 7 patient population among hospitalized patients nationwide [43]. This appears to hold true in many other conflicts as well; the review by Wild et al. likewise demonstrated a predominance of extremity injuries, accounting for one third of injuries overall [7]. Plausibly, this phenomenon is due to the higher survivability of isolated extremity injuries compared to penetrating truncal or head injuries, which is supported by the observation in this study that injuries to the extremities were less likely to have high acuity.

### 4.1 Limitations

This study was subject to five chief limitations. First, due to survival bias, no conclusions can be drawn about the effects of injury mechanism on prehospital acuity, nor about the pattern of injuries occurring in the field. Second, the ongoing attacks compromised prehospital and ED‐level data collection which limited some analyses. In peacetime, MDA routinely collects pre‐hospital data in the form of key demographic and clinical variables that are automatically recorded in the tablets carried by paramedics. However, during the highly disrupted conditions of October 7, MDA personnel had to prioritize evacuation of patients from the scene of injury, including transporting several patients in one ambulance simultaneously. These conditions did not allow for routine data collection practices to be upheld. Therefore, detailed analysis of prehospital data is not possible. Similar deficiencies in data collection occurred at the ED level, especially with respect to patients who were DoA. Given the operational strain posed by the attacks, no provision could be made for more detailed data collection in this group beyond what has been presented in this study. In order to study if these patients were potentially salvageable, autopsies would have been required, which is ethically problematic. Conflicting operational and data collection needs is a recognized challenge in many types of disasters and may be especially pronounced in armed conflicts [44, 45]. Remedial action likely includes automatized data collection, as well as improved system resilience to create sufficient redundancy to enable high‐quality data entry [17]. Third, no provision was made to distinguish between types of blast injury among patients with explosion‐related injuries. Fourth, no Injury Severity Scores or Abbreviated Injury Scores were collected at ED level. While ISS scores are used to assign patient condition for MoH reporting purposes, these are not entered in the ED registry during this process. Information on ISS in the nationwide October 7 patient population has however been published elsewhere [43]. Finally, since the data in this study does not offer information on patients beyond the ED, this limits the understanding of how injury patterns relate to longer‐term outcomes. Many patients who were admitted at BMC were subsequently transferred to other hospitals for further care, where there is no mechanism in place to retroactively reconcile the clinical outcomes in this group with information from the ED registry [46].

## 5 Conclusion

This study has provided a description of the patient population which presented to the hospital closest to the frontline on October 7. Since tactics, technological level, population, infrastructure, and geography vary widely between theaters of conflict, the conclusions from this study are not to be understood as prescriptive; rather, they provide transferrable insights that may be useful to inform preparedness measures elsewhere. Health systems preparing to remain functional in the event of a large, asymmetric attack where national territory is compromised and EMS is denied access may need to anticipate significant prehospital death and that many patients arrive at health care facilities informally. Efforts to build trauma care capacity among lay bystanders, who may access patients before EMS, can be considered to avert preventable deaths. The extensive survival bias seen in this environment may paradoxically result in patients with firearm‐related injuries having more urgent care needs upon reaching hospital than patients with explosion‐related injuries. Given the higher survivability of extremity injuries, these may be overrepresented among patients who reach hospital.

## Author Contributions

**Maximilian P. Nerlander:** conceptualization, methodology, formal analysis, writing – original draft preparation. **Mohammad Abu Issa:** data curation, investigation. **Mor Saban:** conceptualization, supervision, writing – review and editing. **Gili Givaty:** conceptualization, resources. **Yaniv Ovadia:** conceptualization, resources, data curation, supervision, writing – review and editing.

## Funding

Maximilian P. Nerlander's time on the project was funded by the Region Kronoberg Department of Research and Development, Kronoberg, Sweden.

## Ethics Statement

This study was granted ethical approval by the Barzilai Medical Center Helsinki Committee (BRZ‐0083‐24). Ethical approval was also granted by the Swedish National Ethical Review Board (2026‐00449‐02).

## Consent

The authors have nothing to report.

## Conflicts of Interest

The authors declare no conflicts of interest.

## Data Availability

The datasets used and analyzed during the current study are available from the corresponding author on reasonable request.
